# Monoclonal antibodies targeting small airways: a new perspective for biological therapies in severe asthma

**DOI:** 10.1186/s40733-022-00088-2

**Published:** 2022-10-17

**Authors:** Carlo Lombardi, Marcello Cottini, Alvise Berti, Pasquale Comberiati

**Affiliations:** 1 Departmental Unit of Pneumology & Allergology, Fondazione Poliambulanza Istituto Ospedaliero, Via Bissolati, 57, 25100 Brescia, Italy; 2 Poliambulanza Hospital, Via Leonida Bissolati, 57, 25124 Brescia, Italy; 3 Allergy and Pneumology Outpatient Clinic, Bergamo, Italy; 4 Ospedale Santa Chiara and Department of Cellular, Computational and Integrative Biology (CIBIO), University of Trento, Trento, Italy; 5 Santra Chiara Hospital, Largo Medaglie D’Oro, 9, 38121 Trento, Italy; 6 Department of Clinical and Experimental Medicine, Section of Pediatrics, University of Pisa, Pisa, Italy

**Keywords:** Severe asthma, Small airways disease, Biological agents, Omalizumab, Mepolizumab, Benralizumab, Dupilumab, Tezepelumab

## Abstract

Small airway dysfunction (SAD) in asthma is characterized by the inflammation and narrowing of airways with less of 2 mm in diameter between generations 8 and 23 of the bronchial tree. It is now widely accepted that small airways are involved in the pathogenesis of asthma and are a major determinant of airflow obstruction in this disease.

In recent years, specialized tests have been developed, such as Impulse Oscillometry (IOS) and Multiple Breath Nitrogen Washout (MBNW) tests, which have been deemed more accurate in detecting SAD than conventional spirometry. Clinical studies show that SAD is associated with more severe bronchial hyperresponsiveness, worse asthma control, and a higher risk of exacerbations. Recent data from a large cohort study showed that the prevalence of SAD in asthma patients increases with asthma severity. Overall, SAD seems to represent a treatable trait, which makes it appealing for asthma control optimization and exacerbation rate reduction, especially in moderate-to-severe asthma.

Biologic agents are now available for the treatment of different severe asthma phenotypes and endotypes. However, the effect of these therapies on SAD remains poorly characterized. Literature showing that biologic agents can also favorably improve small airway function is accumulating. In particular, anti-IL5 agents (mepolizumab and benralizumab) seems to have a greater impact on SAD as compared to other biological agents, but direct comparisons in prospective randomized controlled trials are lacking.

In this mini-review article, we address the latest evidence on the effect of biological therapies on SAD in patients with severe asthma.

## Introduction

Asthma is a chronic and heterogeneous condition affecting the airways, characterized by inflammatory infiltration and remodelling of the bronchial tree [1]. Even if asthma affects the entire bronchial tree, small airways (those with an internal diameter ≤ 2 mm) have been recognized as the major site of airflow limitation in both asthma and chronic obstructive pulmonary disease (COPD) [2, 3].

Many studies and systematic reviews have suggested that small airway dysfunction (SAD) is associated with more severe bronchial hyper-responsiveness, worse asthma control and a higher number of exacerbations [4–11].

According to the current Global Initiative for Asthma (GINA) guidelines, spirometry remains the method of choice in evaluating respiratory function [12]. However, conventional spirometry reflects mostly the variability and/or the reversibility of airway obstruction and is unable to sensitively evaluate the small airways, becoming abnormal only when approximately 75% of small airways are obstructed [13]. To date, there are numerous diagnostic techniques available to assess SAD, from non-invasive to minimally invasive or invasive, such as spirometry (FEF25–75%, FVC, FVC/SVC), impulse oscillometry (IOS) (R5–R20, X5, ΔX5in-esp, AX, Fres), single breath nitrogen washout (SBNW) or multiple breath nitrogen washout (MBNW) test, body plethysmography (RV, RV/TLC), high-resolution computerized tomography (HRCT), nuclear medicine (scintigraphy, SPECT, PET), 3He-magnetic resonance imaging (MRI), sputum induction, and bronchoscopy [11].

The higher prevalence of SAD, as defined by IOS, in more severe stages of asthma has been confirmed in several real-life studies and the ATLANTIS trial, in which the peripheral abnormalities persisted despite a greater daily dose of inhaled corticosteroids (ICS), suggesting that current therapies have little effect on the airway structural abnormalities, or poor peripheral delivery of inhaled therapy [4, 8, 14–17].

A new personalized approach, termed the “treatable traits” approach, has been suggested to address the limitations of the existing treatment strategies [18, 19].

**Fig. 1:**
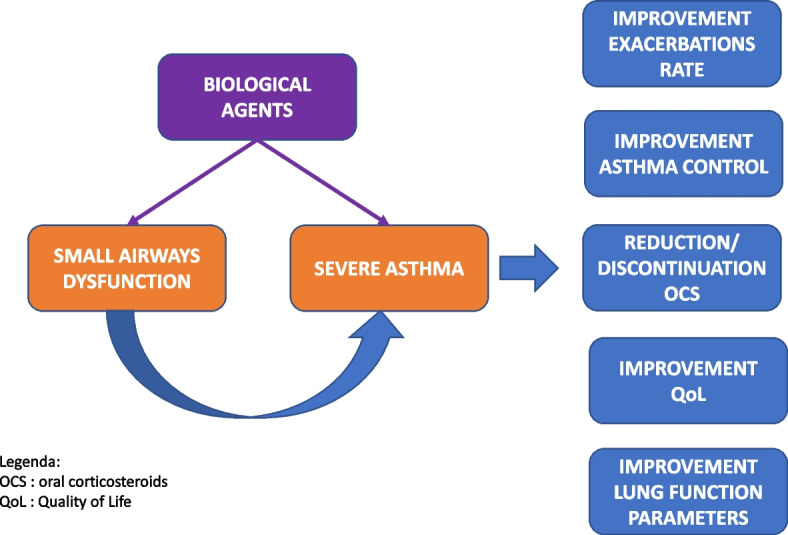
Relationship between severe asthma, small airway disfunction (SAD) and biological agents

From this perspective, SAD appears to possess the characteristics of a treatable pulmonary trait, making it certainly appealing for asthma control optimization and exacerbation rate reduction [11]. This seems especially important in the perspective of moving toward precision medicine. The introduction of biologic agents for the treatment of severe asthma has been shown to be very effective, both in randomized clinical trials and real-life studies, and it is in line with the concept of precision medicine. Therefore, demonstrating that biologic agents can also favorably improve small airway function appears to be of crucial importance (Fig. 1).

In this mini-review article, we address the most recent evidence for the effect of systemic biologic therapies on small airway disease in patients with severe asthma.

### SAD: Prevalence, association with specific asthma phenotypes and poor asthma control

The prevalence of SAD in asthma appears to be very high, likely 50 percent and above based on studies to date [20]. In the ATLANTIS study, the largest multinational study showing the contribution of SAD to asthma severity, 91% of asthmatics were found to have SAD, which was strongly present across all GINA severity steps [4].

SAD has been associated with some clinical phenotypes, such as active smokers, elderly patients with long-standing asthma and presence of fixed airflow obstruction, patients with nocturnal and exercise-induced symptoms, and severe/uncontrolled asthma [6, 7, 9]. In more recent real-life studies, multivariable analyses, classification tree analysis and structural equation modeling indicated that exercise-induced symptoms, overweight/obesity, asthma-related nocturnal symptoms, older age, smoking, and type-2 inflammation are strong independent predictors of SAD in patients with community-managed asthma [8, 21–23]. These features may help identify SAD among patients with asthma, especially when IOS cannot be performed.

The identification of SAD is of particular importance since it is strongly associated with worse asthma control. In the ATLANTIS study, SAD (identified by both IOS and spirometry) was significantly associated with asthma control, history of exacerbation, and disease severity [4], confirming preliminary data from real-life studies [8]. Kraft and colleagues recently published the longitudinal one-year follow-up data of the ATLANTIS study, which showed that SAD was longitudinally associated with poor asthma control, exacerbations and quality of life [5].

These results are consistent with other published data, in which asthma control was closely related to SAD and clinical phenotypes associated with SAD [8, 11, 21–27]. Notably, in the majority of these studies, the IOS is better associated with asthma control than spirometry, supporting the importance of using IOS and more modern tools in addition to spirometry in the evaluation of asthma control.

### SAD in severe asthma

The chronic inflammation which characterizes asthma involves the entire lung, from the large proximal to the small distal airways. The involvement of the small airways contributes to the morbidity of asthma, particularly severe asthma. Challenges to implementing small airway assessments in the routine clinical setting and as part of severe asthma management include technical aspects of assessment and monitoring of SAD, and the impact of small airways on asthma therapeutic delivery and outcomes [28]. About 5–10% of patients with asthma are deemed to have a severe disease [29], which represents up to 50% of total asthma-related healthcare costs. Severe asthma can be divided into several pheno/endotypes, of which severe eosinophilic asthma is among the most studied [30]. Autopsies from fatal asthma cases showed intense inflammation, small airway structure abnormalities and luminal plugging in peripheral airways [31, 32].

Despite evidence of an association between SAD and neutrophilic airway inflammation [33], it was recently demonstrated that eosinophilic rather than neutrophilic airway inflammation is the main driver of SAD in asthma [34].

In the ATLANTIS study, SAD was strongly present across all GINA severity stages but consistently higher in more severe asthma (GINA step 5), similar to findings of previous studies [8, 15, 21].

The advances in the knowledge of different phenotypes and endotypes of severe asthma have led to very innovative therapies, such as biological agents for severe asthma. These medications are mostly directed against cytokines and cells involved in the type-2 inflammatory pathway, thus modifying the natural course of the disease by reducing airway inflammation without the collateral damage associated with the use of systemic corticosteroids. In this context, demonstrating that biologic agents can also favorably improve small airway function appears to be of crucial importance. In particular, it seems prospectively important to achieve modulation by biological agents of the activity of eosinophils at the level of the distal airways.

### SAD and anti-IgE agents (omalizumab)

Omalizumab, a humanized recombinant monoclonal antiIgE antibody, inhibits the binding of serum IgE to the FcεRI receptors on mast cells and basophils which reduces the inflammatory response caused by the activation of such effector cells when interacting with the allergen [35]. Omalizumab has also been shown to have a preventative effect on viral-induced exacerbations in children with allergic asthma by reducing susceptibility to rhinovirus infections [36]. Many trials have demonstrated that omalizumab significantly decreases the number of severe exacerbations, the dose amounts of inhaled and/or oral corticosteroid, and improves the quality of life in children, adolescents, and adults with severe allergic asthma [37, 38].

Regarding the impact of omalizumab on SAD, some works have evaluated this aspect with contradictory results (Table 1).

**Table 1:** Biological agents and small airway dysfunction (SAD): summary of the main clinical studies performed

Biological agent	Authors (reference)	Patients treated	Diagnostic tool to detect SAD	Main Results or Endpoints	Impact on SAD
OMALIZUMAB	Huang et al. [39]	124	FEF 25–75%	improvement in small airway ventilation	+ + +
	Tajiri et al. [40]	26	CT scan FEF 25–75%	airway-wall thickness reduced signi-ficantly 48 weeks of omalizumab treatment	+ +
	Paganin et al. [41]	207	forced vital capacity (FVC), pre- and postbronchodilator FEV1, residual volume (RV), and total lung capacity (TLC)	After omalizumab, FEV1 improved at 6 months in responder patients and then remained stable for 2 years. RV and RV/TLC improved at 6 months	+
	Saadeh et al. [42]	12	IOS, spirometry	IOS indices were significantly improved after 3 to 4 months of treatment; but were not paralleled by changes in FEV1	+ +
OMALIZUMAB + MEPOLIZUMAB + BENRALIZUMAB	Chan et al. [43]	Omalizumab [20], mepolizumab [30], benralizumab [6]	IOS, spirometry	FEF25-75% increased significantly in the omalizumab sub-group(15 of 20 patients) but not significantly in the anti-IL5 subgroup (21 of 36 patients). Insufficient evaluable IOS data to perform a meaningful analysis	+ +
MEPOLIZUMAB	Antonicelli et al. [44]	18	Forced Oscillation Technique (FOT)	Statistically FOT parameters improvement after mepolizumab	+ + +
	Sposato et al. [45]	134	spirometry	FEV1% and FEF25-75% improved significantly after mepolizumab	+ + +
	Farah et al. [46]	20	spirometry, FeNO, multiple breath nitrogen washout to measure Lung Clearance Index, regional ventilation inhomogeneity in acinar (Sacin) and conducting (Scond) airways	Early improvement in small airway function has been associated with asthma control	+ +
	Yilmaz et al. [47]	41	spirometry	not found significant changes in FEV1 and FEF25-75 values at baseline, 12th, 24th, and 52nd weeks	No impact
	Maglio et al. [48]	105	spirometry	significant improvement in FEF25-75% was demonstrated	+ + +
DUPILUMAB + MEPOLIZUMAB + BENRALIZUMAB	Abdo et al. [49]	mepolizumab [18], benralizumab [1], dupilumab [1]	IOS, FEF25-75%, air trapping (RV and RV/ TLC)	Responders had significantly higher baseline FDR (frequency dependence of resistance) compared to partial or non-responders but similar FEV1, FEF25–75, RV and RV/TLC	+ +
BENRALIZUMAB	Panettieri et al. [50]	118	spirometry/whole-body plethysmography	Improvements in FEF25–75% observed for the benralizumab-treated group (but not significant). Improvements in hyper-inflation indices and IC were greater for pts receiving benralizumab vs placebo	+ +
	Badal et al. [51]	12	spirometry, forced oscillation technique (FOT), multiple breath nitrogen washout (MBNW)	significant improvements in Sacin, X5, and a trend for improvement in Scond	+ + +
	Mcintosh et al. [52]	27	spirometry, IOS, FeNo	X5 and AX were significantly different after benralizumab across subgroups, and both X5 and AX were significantly different after 28-days of benralizumab in the FeNO high subgroup. Clinically relevant ventilation defect percent (VDP) improvements were also observed in the FeNO intermediate and FeNO high sub-groups	+ + +
DUPILUMAB	Castro et al. [53]	1264	spirometry / FEF25-75%	Improvement not limited to the large airways but also extends to the small airways	+ +
	Rabe et al. [54]	103	spirometry /FEF25-75%	At Week 24 in the overall population, pre- and post-BD FEV1, FVC, FEV1/FVC and FEF25–75% had improved vs placebo Improvements were early and generally sustained over 24-weeks treatment	+ +
	Pelaia et al. [55]	20	spirometry/whole-body plethysmography, FeNO	Dupilumab induced a significant reduction in lung hyperinflation, significant increase in median FEF25–75%	+ + +
	Minagawa et al. [56]	62	spirometry,forced oscillation technique (FOT), FeNO	FEV1, %FEV1, %FVC, improved significantly after three months of dupilumab treatment. FeNO was markedly decreased.. R5, R20, X5, and Fres were significantly correlated with FEV1 before and three months after dupilumab administration	+ + +
	Bacharier et al.[57]	236	Spirometry/FEF25-75%	significant improvements in percent predicted pre-bronchodilator FEV1 and FEF25-75%	+ + +

Legend: No impact/+/++/+++ indicate the level of impact of the biologic agent studied in every manuscript on SAD. This is a semiquantitative scale that took into account the strongness of the associations/correlations, the numbers of associations or correlation in the single paper (i.e. more than one significant associations, as spirometry values improvement + IOS improvement are weighted more than improvement only in IOS or spirometry), the number of patients of the cohort, etc.

Huang et al. evaluated the long-term effectiveness of omalizumab in adult patients with severe allergic asthma comparing continuous treatment versus boosting treatment [39].

Of the 124 patients treated, a significant reduction in annual exacerbations and improvement in SAD evaluated by FEF25–75% were found in the continuation group (*n* = 110). In contrast, the boost group (*n* = 14) had significantly increased annual exacerbations, impaired small airway function, and worse asthma control, suggesting that continuous omalizumab treatment is preferable.

In another study conducted by Pasha et al., omalizumab did not lower the alveolar NO concentration (CalvNO), which could reflect small airway inflammation. However, the authors concluded that the model they used may not have been sufficiently sensitive to detect small changes in CalvNO [58].

In another study conducted by Taijri et al. the aim was to comprehensively evaluate the efficacy of omalizumab, including its effects on small airways and airway remodeling, in adult patients with severe refractory asthma. Assessment by computed tomography revealed that airway-wall thickness reduced significantly after 48 weeks of omalizumab treatment (no significant changes were found at 16 weeks). Instead, FEF 25%–75% was not influenced by omalizumab, but only a small sample of patients was evaluated [40].

In a multicenter analysis over 2 years, the authors found a decrease in the RV and RV/TLC ratio at 6 months but no significant change in TLC in patients treated with omalizumab. After 6 months, RV worsened slightly but RV/TLC remained stable. RV is higher in patients with air trapping, a phenomenon observed in part in peripheral airway alterations. Interestingly, a greater RV improvement was observed in patients with severe airway obstruction (FEV1 ≤ 50%) than in those with less obstruction. These patients may have lung hyperinflation that could reflect air trapping and small airway obstruction.

Saadeh et al., evaluated twelve moderate-to-severe asthmatic patients in an open-label safety study of omalizumab using IOS, in addition to spirometry [42]. IOS measures included low-frequency resistance (R at 5 Hz, R5), and low frequency integrated reactance (AX). IOS indices were significantly improved after 3 to 4 months of treatment but were not paralleled by changes in spirometry (FEV1). The conclusion was that low-frequency FO indices provide objective evidence of omalizumab efficacy, while spirometry does not yield such objective evidence.

Chan et al. recently performed a mixed cohort study treating 20 of 56 severe asthmatics with omalizumab (the other patients were treated with mepolizumab (30 pts) and benralizumab (6 pts), respectively) and evaluating SAD with spirometric parameters and IOS (see also mepolizumab and benralizumab section) [43]. In the overall group, FEF25–75% but not FEV1% improved significantly pre- versus post-biologic therapy. In the SAD subgroup defined by R5-R20 ≥ 0.08 kPa/L/s (*n* = 15), R5-R20 but not AX improved significantly pre- versus post-biologic therapy. Of these, 11 patients received anti-IL5 therapy with the remaining receiving omalizumab. In patients with pre-biologic FEF25–75 < 60% (*n* = 43) there were significant improvements in the Asthma Control Questionnaire (ACQ) score and OCS-requiring exacerbations, but no differences were observed in FEV1 or IOS. Notably, 10 patients with R5-R20 ≥ 0.08 kPa/L/s also had a pre-biologic FEF25–75 < 60%. When comparing patients according to biologic therapy received, FEF25–75% increased significantly in the omalizumab subgroup (15 of 20 patients) but not significantly in the anti-IL5 (mepolizumab and benralizumab) subgroup (21 of 36 patients). In their conclusions, the authors suggested that forced expiratory flow between 25 and 75% of the forced vital capacity should be incorporated to assess the improvement of SADin patients with severe asthma taking biological drugs. IOS may be useful as an adjunct, particularly in patients with SAD defined as IOS at baseline.

### SAD and anti-IL-5 agents (mepolizumab, reslizumab, benralizumab)

#### Mepolizumab

Mepolizumab is a humanized monoclonal antibody directed against IL-5 (anti-IL5). Several randomized controlled trials in adults and adolescents with severe asthma have shown the efficacy of mepolizumab in reducing blood eosinophilia. This, in turn, lowers the rate of severe exacerbations and the usage of oral corticosteroid while improving asthma controls and increasing lung function (Table 1) [59].

In 2019 Farah et al. evaluated a prospective cohort of 20 adults with severe eosinophilic asthma treated monthly with mepolizumab [46]. Symptom control improved rapidly after commencing mepolizumab in patients with severe eosinophilic asthma. Early improvement in small airway function has been associated with asthma control and may contribute significantly to therapeutic response, according to the authors.

Abdo et al. studied 20 patients with severe eosinophilic asthma under treatment with anti-type 2 agents (mepolizumab, *n* = 18; benralizumab, *n* = 1; dupilumab, *n* = 1) [49].

Responders had a significantly higher baseline frequency dependence of resistance (FDR, R5-20) derived from IOS compared to partial or non-responders but similar FEV1, FEF25–75, air trapping (measured as RV and RV/TLC). The main finding of this study was that SAD improves substantially under anti-T2 biological therapy in patients with severe eosinophilic asthma. Furthermore, pre-treatment IOS measures of SAD demonstrated to be meaningful predictors of clinical response, thereby indicating that severe SAD might describe a distinct phenotype with therapeutic implications among patients with severe eosinophilic asthma. Similar data and conclusions were obtained by Antonicelli et al. who performed a prospective study using Forced Oscillation Technique (FOT) to evaluate the effect of mepolizumab on SAD on 18 patients with severe eosinophilic asthma [44]. The authors state that treatment-induced changes in peripheral airway function could be effectively monitored by FOT and could become an objective method to assess response to mepolizumab, in addition to current continuation rules.

Sposato et al. retrospectively analyzed 134 adult severe asthmatics, treated with mepolizumab for at least 6 months (mean duration:10.9 ± 3.7 months) [45]. FEV1% improved significantly after MEP. Mean FEF25-75 also increased from 37.4 ± 25.4% to 47.2 ± 27.2% (*p* < 0.0001). Mepolizumab treatment also led to a significant ACT improvement and exacerbations significantly reduced. Therefore, in real life, mepolizumab significantly improved all outcomes including small airway obstruction, suggesting its possible role also in distal lung region treatment.

Yilmaz et al. retrospectively analyzed 41 patients with severe eosinophilic asthma who were receiving fixed-dose mepolizumab [47]. Mepolizumab significantly reduced asthma exacerbation rates, reduced OCS dose, and improved ACT scores at 12th, 24th, and 52nd weeks. However, no significant changes were found in FEV1 and FEF25–75 values at baseline, 12th, 24th, and 52nd weeks. In a recent real-life study, a significant improvement in FEF25–75% was demonstrated in 105 patients with severe eosinophilic asthma [48]. FE25–75% values showed a highly significant, gradual, and persistent increase (from 32.7 ± 18.2% at baseline to 48.6 ± 18.4% after 18 months). The latter data were also further confirmed by Chan et al. who evaluated a total of 56 patients with severe refractory asthma treated with omalizumab (20 patients), mepolizumab (30 patients), and benralizumab (6 patients), respectively [43]. Patients with severe refractory asthma with relatively well-preserved baseline FEV1% experienced significant improvements in small airway function measured by FEF25–75% and by IOS-defined SAD (R5-R20 ≥ 0.08 kPa/L/s) with clinical response to biologic therapy.

To date, mepolizumab appears to be the most studied biological agent in this area which also highlights a significant effect of this anti-IL-5 biological on SAD.

#### Reslizumab

Reslizumab is a humanized monoclonal antibody anti-IL5, resulting in a reduction of sputum eosinophils and blood eosinophils and, in turn, reduction of exacerbations and asthma symptoms and improved lung function [60]. To our knowledge, no studies published to date have specifically evaluated the impact of reslizumab on SAD.

#### Benralizumab

Benralizumab is a monoclonal antibody of murine origin that binds the alpha chain of the IL-5 receptor (anti-IL5R) leading to antibody-dependent cell-mediated cytotoxicity and almost complete depletion of eosinophils in the bone marrow, blood and peripheral tissues [61]. Several studies assessed the effect of benralizumab on SAD (Table 1).

Panettieri et al. performed a multicenter, randomized, double-blind, parallel group, placebo-controlled, phase IIIb study (SOLANA) with the administration of benralizumab (*n* = 118) versus placebo (*n* = 115) [50]. Treatment with benralizumab resulted in a non-statistically significant improvement from baseline in lung function over the maintenance period, as assessed by pre-BD FEV1, FVC, and whole-body plethysmography, for patients with severe eosinophilic asthma and previous asthma exacerbations, non-statistically significant improvements in FEF25–75% were also observed at all time points for the benralizumab-treated group. However, no improvements reached nominal significance compared with placebo. Improvements in hyperinflation indices and IC were greater for patients receiving benralizumab versus placebo. The observed early changes in lung volume suggest that the anti-inflammatory effect of benralizumab may be manifested as deflation over time for patients with hyperinflation, who potentially have a greater degree of airway remodeling and may represent a favorable effect on SAD.

More convincing data on the impact of benralizumab on SAD come from the study by Badal et al. [51]. There were significant improvements in Sacin, X5) and a trend for improvement in Scond. At week 4, the change in ACQ-5 correlated with a change in FEV1, Scond, R5, and X5. At baseline, physiological indices were more predictive of symptom improvements compared to eosinophil count. In conclusion, small and large airway function improved soon after commencing benralizumab and predicted symptom improvement was reported by patients.

Finally, Mcintosh et al. evaluated 27 patients with eosinophilic asthma before and after treatment with benralizumab and hypothesized that those with baseline FeNO > 50 ppb would report significant oscillometry and ventilation defect percent (VDP) responses, and this would not be observed in participants with normal baseline FeNO [52]. The oscillometry measurements of elastance at 5 Hz (X5) and reactance area (AX) were significantly different post-benralizumab across subgroups, and both X5 and AX were significantly different after 28 days of benralizumab in the FeNO high subgroup. Clinically relevant VDP improvements were also observed in the FeNO intermediate (Δ =—5%) and FeNO high (Δ =—6%) subgroups only. The authors concluded that the level of baseline FeNO predicted significant oscillometry and MRI VDP responses to benralizumab, 28 days post-therapy.

#### SAD and anti-IL-4/IL-13 (dupilumab)

Dupilumab, is a fully human monoclonal antibody that binds to the alpha subunit of the IL-4 receptor (mutual to IL-4 and IL-13 receptors), thereby inhibiting both the IL-4 and IL-13 pathway [62].

In patients with severe asthma, dupilumab reduces severe exacerbations and the use of oral corticosteroids. In addition, it significantly improves the quality of life, symptom control and lung function parameters (Table 1) [63, 64].

We have interesting experimental and clinical data on the role of dupilumab in SAD. Manson et al. performed an experimental study in human small bronchi with the aim of providing insight into which of type 2 and type 17 cytokines causes hyperresponsiveness of airway smooth muscle [65]. Explanted small bronchi isolated from human lung tissue and human airway smooth muscle cells were treated with 100 ng/mL of IL-4, IL-5, IL-13, or IL-17A, and contractile responses, Ca2^+^ mobilization, and receptor expression were assessed. The glucocorticoid-insensitive hyper-responsiveness in isolated human airways induced by IL-13 and IL-4 provides evidence that the IL-4Ra pathway should be targeted as a new strategy for the treatment of airway hyperresponsiveness in small airways.

Castro et al. performed an analysis of pre-specified secondary and post hoc results from patients with uncontrolled, moderate-to-severe asthma enrolled in the LIBERTY ASTHMA QUEST phase 3 trial). In adults with uncontrolled persistent asthma, dupilumab administration was associated with significant improvements across a range of lung-function measures including FEV, FVC, and FEF25–75%, suggesting that the observed improvement is not limited to the large airways but also extends to the small airways [53].

Rabe et al. evaluated the impact of dupilumab on lung function parameters in patients with oral corticosteroid-dependent severe asthma [54]. At Week 24 in the overall population, pre- and post-BD FEV1, FVC, FEV1/FVC and FEF25–75% had improved versus placebo. Improvements were early and generally sustained over a 24-week treatment.

In a single-center observational study, Pelaia et al. evaluated in a real-life setting the short-term therapeutic effects of dupilumab in patients with severe asthma and nasal polyposis [55]. Dupilumab induced a significant reduction in lung hyperinflation caused by airflow limitation. In comparison to baseline, 4 weeks after the first administration, median RV had significantly diminished. RV reduction was associated with a concomitant decrease in TLC. In addition to such rapid and impressive deflating effects, it was also observed that dupilumab had improved small-airway obstruction, with a significant increase in median FEF25–75% from 1.47 ± 0.85 L/sec to 1.80 ± 0.86 L/sec (*p* < 0.01). Evaluated together, these effects on respiratory functional parameters mentioned above may be indicative of a favorable and rapid dupilumab effect on SAD.

Minagawa et al. retrospectively evaluated the effects of dupilumab in 62 patients who received dupilumab for eosinophilic sinusitis comorbid with asthma at a single center in Japan [56].

FEV1, %FEV1, %FVC, treatment steps for asthma and ACT improved significantly after three months of dupilumab treatment. FeNO was markedly decreased, whereas IgE and eosinophil counts showed no significant changes. Pre- and post-treatment respiratory resistance (Rrs) and respiratory reactance (Xrs) correlated significantly with FEV1. For the first time, these authors monitored the effects of dupilumab using FOT in combination with spirometry and evaluated the correlation between the two modalities. R5, R20, X5, and Fres were significantly correlated with FEV1 before and three months after dupilumab administration, suggesting that both modalities can be useful for monitoring efficacy. Furthermore, the improvement rate of R5 after three months of treatment with dupilumab in patients with good FEV1/FVC was significantly higher than that of FEV1. This study demonstrated that dupilumab not only has a marked effect on CRSwNP but may also improve respiratory parameters related to SAD.

Bacharier et al. studied the effect of dupilumab on lung function in children aged 6 to 11 years with uncontrolled, moderate-to-severe asthma and a T2 inflammatory asthma phenotype [57]. In this phase 3, double-blind, placebo-controlled LIBERTY ASTHMA VOYAGE study (NCT02948959), add-on dupilumab demonstrated significant improvements in percent predicted pre-bronchodilator FEV1 and FEF25–75%.

### SAD and anti-TSLP (tezepelumab)

In December 2021**,** tezepelumab was approved by the FDA for the treatment of severe asthma, and is the only biologic approved for severe asthma with no phenotype (e.g. eosinophilic or allergic) or biomarker limitation within its approved label [66]. Tezepelumab is an IgG2 monoclonal antibody that targets thymic stromal lymphopoietin (TSLP), preventing its interaction with the heterodimeric TSLP receptor, and impacting both type-2 and non-type 2 inflammatory pathways, including allergic, eosinophilic inflammation, and airway hyperresponsiveness. Based on our research, no specific studies have evaluated the impact of tezepelumab on small airway function; but many interesting findings derive from the CASCADE study. Indeed, Diver et al. investigated the mechanism of action of tezepelumab by assessing its effects on airway inflammatory cells, airway remodeling, and airway hyperresponsiveness, and indirectly may lead us to believe that favorable action on small airways is possible by tezepelumab [67].

CASCADE was an exploratory, double-blind, randomized, placebo-controlled, parallel-group, phase 2 study enrolling patients aged 18–75 years with uncontrolled, moderate-to-severe asthma. The primary endpoint was the change from baseline to the end of treatment in the number of airway submucosal inflammatory cells in bronchoscopic biopsy samples. Airway remodeling was assessed via the secondary endpoints, including airway hyperresponsiveness to mannitol. Treatment with tezepelumab resulted in a nominally significantly greater reduction from baseline to the end of treatment in airway submucosal eosinophils versus placebo (ratio of geometric least-squares means 0·15 [95% CI 0·05–0·41]; nominal *p* < 0·0010), with the difference seen across all baseline biomarker subgroups. There were no significant differences between treatment groups in the other cell types evaluated (ratio of geometric least-squares means: neutrophils 1·36 [95% CI 0·94–1·97]; CD3 + T cells 1·12 [0·86–1·46]; CD4 + T cells 1·18 [0·90–1·55]; tryptase + mast cells 0·83 [0·61–1·15]; chymase + mast cells 1·19 [0·67–2·10]; all *p* > 0·10). In the assessment of secondary endpoints, there were no significant differences between treatment groups in reticular basement membrane thickness and epithelial integrity. The reduction in airway hyperresponsiveness to mannitol was significantly greater with tezepelumab versus placebo (least-squares mean change from baseline in interpolated or extrapolated provoking dose of mannitol required to induce ≥ 15% reduction in FEV1 from baseline: tezepelumab 197·4 mg [95% CI 107.9 to 286.9]; placebo 58.6 mg [-30.1 to 147.33]; difference 138.8 [14.2 to 263.3], nominal *p* = 0.030).

## Conclusions

Biologic therapies for severe asthma can lead to improvements in asthma control, OCS use, and exacerbation frequency. Emerging data show that biological agents, in particular anti-IL5 agents (mepolizumab and benralizumab), can positively impact also on SAD, even though direct comparisons in prospective randomized controlled trials are lacking. However, data on the effect of biologics on SAD remain limited; which biological agents is the best to control SAD, how the best treatment for every situation or patient should be chosen, for how long treatment should be done and, eventually, when withdraw the treatment and what happen to SAD after that moment have not been addressed yet.

Given the clinical impact of SAD on asthma control and severity, it seems advisable to have SAD actively checked as part of the daily management of patients with severe asthma. Severe SAD may represent a distinct phenotype of severe eosinophilic asthma that substantially improves under anti-type-2 biological therapy. Measures of small airway function with both spirometry and other non-invasive tools, such as IOS, might be useful in selecting appropriate patients who qualify for anti-type-2 biological therapy in addition to blood eosinophil count. Clinical trials involving larger cohorts and multimodular diagnostic assessment of small airway function are needed to confirm the added value of SAD as a prognostic marker of response to a particular biological agent. 

## Data Availability

Upon request.
